# Normative data for the 6-min walk test in 11–14 year-olds: a population-based study

**DOI:** 10.1186/s12890-021-01666-5

**Published:** 2021-09-21

**Authors:** Mario Kasović, Lovro Štefan, Vilko Petrić

**Affiliations:** 1 Department of General and Applied Kinesiology, Faculty of Kinesiology, University of Zagreb, Horvaćanski zavoj 15, Zagreb, Croatia; 2 Department of Sport Motorics and Methodology in Kinanthropology, Faculty of Sports Studies, Masaryk University, Brno, Czech Republic; 3 Department of Educational Studies, Faculty of Teacher Education, University of Rijeka, Rijeka, Croatia; 4 Recruitment and Examination (RECETOX), Faculty of Science, Masaryk University, Brno, Czech Republic

**Keywords:** Pediatric, Exercise capacity, Standards, Testing

## Abstract

**Background:**

The 6-min walk test (6MWT) has become an established measure for assessing exercise capacity in children with chronic diseases. However, little evidence has been provided regarding population-based normal data in healthy children. The main purpose of the study was to provide normative data in a large sample of children.

**Methods:**

In this cross-sectional study, 4352 children between 11 and 14 years were recruited (66% girls). The main outcome measure was the distance walked for six minutes. Sex- and age-specific percentile values (5th, 15th, 25th, 50th, 75th, 85th and 95th) for the 6MWT were created and the differences and correlations were examined by the analysis of variance and Pearson’s coefficient of correlation.

**Results:**

The mean distance walked in 6 min was 576 ± 93 m in boys and 545 ± 92 m in girls, respectively. The mean walking speed for boys and girls was 98 ± 5 m/min and 91 ± 6 m/min. Older boys and girls performed better, compared to their younger counterparts (*p* for age < 0.001). The 6MWT was significantly correlated with age (*r* = 0.24, *p* < 0.001), height (*r* = 0.09, *p* < 0.001), weight (*r* =  − 0.13, *p* < 0.001) and body-mass index (*r* =  − 0.26, *p* < 0.001).

**Conclusions:**

This is the first population-based study aiming to provide normative data for the 6MWT in healthy children between 11 to 14 years. Children in lower percentiles are ‘target groups’ for special intervention aiming to enhance the performance.

## Background

The six-minute walk test (6MWT) has become a widely used measure for assessing functional exercise capacity at submaximal level [1, 2]. It is described as the distance person can walk at self-paced speed over the period of 6 min [3–5]. Previous evidence highlights the importance of using the 6MWT, in terms of its safety, reliability and validity properties, and usefulness in assessing functional assessment and is a powerful prognostic marker for functional capacity in both healthy and unhealthy individuals [6]. Factors associated with shorter and longer 6-min distance have been described previously [6]. The 6MWT has been shown as inexpensive and safe tool [6, 7], often associated with mortality risks in patients with chronic respiratory diseases [8, 9], cystic fibrosis [10] and hypertension [11].

In the past two decades, the 6MWT has been increasingly used in children with chronic diseases [10, 12–15]. Although its practical implications have been well-documented [16], little evidence has been provided for normative data in healthy children [17]. Specifically, a study by Cacau et al. [17] has identified twelve studies establishing the 6MWT standards. The reference values reviewed in the study were very heterogenous, particularly because of small sample size and different settings [18–23]. Thus, the findings obtained from the aforementioned studies may not be applicable for the population of children in other countries. Thus, both country-specific and worldwide normative values for the 6MWT should be generated, for the purposes of tracking annual changes of functional status within the country and to compare the data between the countries. Since studies have shown a great range in walking distance [17], each country should implement a study to determine normative values for the 6MWT, considering sociodemographic, anthropometric and other cultural differences. The newly established data for the 6MWT would help health-related professionals and physical education teachers for initial screening of functional exercise capacity and detecting those individuals at higher risk of ‘poor’ performance, from which special interventions and policies aiming to enhance functional performance can be implemented within the school system.

Therefore, the main purpose of the study was to create sex-specific and age-specific normative data for the 6MWT in a large sample of children.

## Methods

### Study participants

In this observational study, we approached to children between ages 11 and 14 years (50.8% girls) from randomly selected 10 primary schools located in the city of Zagreb. To be included, children had to be healthy and participated in physical education classes at the time of study and had to be not specifically trained for performance in the administered tests. According to the Croatian Bureau of Statistics for the year 2020 [24], there were 29 358 children aged between 11 and 14 years in total. Our sample size was estimated to be 3730 by using 95% confidence level, 1.5% margin error and the significance of *p* < 0.05. At the beginning, we recruited 4625 children. After the initial screening, 163 were absent and 110 did not attend physical education classes when the tests were being administrated, because of personal issues. Our final sample was comprised of 4352 children (mean age ± SD = 12 ± 1 years, mean height = 156 ± 10 cm, mean weight = 47 ± 11 kg, mean body-mass index = 19 ± 3 kg/m^2^; 66% of girls). Before the testing, physical education teachers responsible for undertaking the tests in each school were briefly instructed about the measurement procedures. The study was approved by the Faculty of Kinesiology, University of Zagreb, Croatia. Parent of each participant and all participants gave informed written consent before enrollment into the study. Analyses and procedures performed in the study were anonymous and conducted in accordance with the Declaration of Helsinki regulations.

### 6MWT

The 6MWT was used to assess functional capacity of the children. The test was conducted using a 30-m straight corridor with a flat, firm ground and with two cones placed at each end of the course. We followed the testing procedure from the ATS Committee guidelines [16]. Before performing the test, the children were well-rested and instructed to walk around the measured lap. If a child had any kind of problem during the test (respiratory or locomotor issues, fatigue), they were told to slow down or stop the test. Children were divided into small groups of five to perform the test, to prevent from competition [2]. The final score was expressed in distance covered in meters (m) during a 6-min period. In addition, we calculated the mean speed of each child by dividing the final score by 6 [6-min distance covered (m)/6].

Height and weight were objectively measured using portable stadiometer and digital scale with a precision of 0.1 mm and 0.1 kg. Body-mass index was calculated by using the following formula: weight (kg)/height (m^2^). Age was self-reported.

### Data analysis

Basic descriptive statistics are presented as mean and standard deviation (SD). The Kolmogorov–Smirnov tests showed that data were normally distributed. Sex and age differences were calculated by using analysis of variance (ANOVA) with post hoc comparison test between the groups. To calculate correlations between all the study variables, we used Pearson coefficient of correlation (*r*). All the assumption, including the level of Leven’s test of homogeneity, normal population distribution and data independency were met. Multiple regression analysis was performed, to examine the associations between sex, age, height and weight (the independent variables) with the 6MWT (the dependent variable). Standardized beta coefficient of correlation (*β*) and the coefficient of determination *R*^2^ were used to present the associations. For each variable, we determined sex- and age-specific percentile values (5th, 15th, 25th, 50th, 75th, 85th and 95th) and used the Lambda (L), Mu (M) and Sigma (S) method, in which the optimal power to obtain normality is summarized by a smooth (L) curve and trends in the mean (M) and coefficient of variation (S) are similarly smoothed. Next, all three curves (L, M and S) are summarized based on the power of age-specific Box–Cox power transformations for normalizing the data [25]. The LMS method assumes that the data can be normalized by using a power transformation and removing the skewness. For correlation and multiple regression analyses, we calculated the 95% confidence interval (95% CI) with the significance level of *p* < 0.05. All analyses were performed in Statistical Packages for Social Sciences version 24. (SPSS Inc., Chicago, Illinois, USA).

## Results

Basic descriptive statistics are presented in Table 1. Boys were taller, heavier and had higher body-mass index values, compared to girls. The mean distance walked in 6 min was 576 ± 93 m in boys and 545 ± 92 m in girls, respectively. The mean walking speed for boys and girls was 98 ± 5 m/min and 91 ± 6 m/min.

**Table 1:** Basic descriptive statistics of the study participants (*N* = 4352)

Study variables	Total (*N* = 4352)	Boys (*N* = 1471)	Girls (*N* = 2881)	*p* for sex
	Mean ± SD	Mean ± SD	Mean ± SD	
Age (years)	12 ± 1	12 ± 1	12 ± 1	0.890
Height (cm)	156 ± 10	159 ± 11	155 ± 10	< 0.001
Weight (kg)	47 ± 11	49 ± 13	46 ± 10	< 0.001
Body-mass index (kg/m^2^)	19 ± 3	19 ± 3	19 ± 3	0.756
6MWT (m)	561 ± 93	576 ± 93	545 ± 92	< 0.001
Speed (m/min)	94 ± 5	98 ± 5	91 ± 6	< 0.001

*p* < 0.05

The 6MWT was significantly correlated with age (*r* = 0.24, 95% CI 0.21–0.26, *p* < 0.001), height (*r* = 0.09, 95% CI 0.06–0.12, *p* < 0.001), weight (*r* =  − 0.13, 95% CI − 0.16 to − 0.10, *p* < 0.001) and body-mass index (*r* =  − 0.26, 95% CI − 0.29 to − 0.23, *p* < 0.001). Multiple regression analysis showed that sex (unstandardized *β coefficient* =  − 0.81.7, 95% CI − 92.8 to − 70.5, *p* < 0.001), age (unstandardized *β coefficient* = 62.0, 95% CI 56.0–68.1, *p* < 0.001), height (unstandardized *β coefficient* = 3.0, 95% CI 2.1–3.8, *p* < 0.001) and weight (unstandardized *β coefficient* =  − 7.5, 95% CI − 8.2 to − 6.8, *p* < 0.001) entered simultaneously into the model were significantly associated with the 6MWT (*R* = 0.44, *R*^2^ = 19%, standard error of estimates = 168.5 m, *p* < 0.001).

Normative values for the 6MWT are presented in Table 2. A significant rising trend in the 6MWT in both sexes was observed. Significant differences between sex (*F*_1,7_ = 144.05, *p* < 0.001), age (*F*_3,7_ = 92.68, *p* < 0.001) and sex*age interaction (*F*_3,7_ = 5.50, *p* < 0.001) were observed. Specifically, boys achieved better walking distance, compared to girls (mean difference = 69.2 m, *p* < 0.001), older boys and girls performed better, compared to their younger counterparts (11-year-olds = 526.7 m; 12-year-olds = 552.5 m; 13-year-olds = 573.6 m and 14-year-olds = 592.3 m; *p* < 0.001 for all age groups) and boys in one age group performed better, compared to girls in the same age group (*p* < 0.001).

**Table 2:** Normative data for the 5th, 15th, 25th, 50th (median), 75th, 85th and 95th percentile of the 6MWT in the study participants (*N* = 4352)

Measure	Sex	Age	N	P5	P15	P25	P50	P75	P85	P95
6MWT (m)	Boys	11	404	385	464	490	560	605	630	665
		12	387	388	465	503	570	630	660	700
		13	385	420	490	527	595	653	675	723
		14	295	452	525	565	630	680	708	750
	Girls	11	675	349	420	455	512	570	576	630
		12	767	391	450	495	540	600	630	690
		13	732	420	462	500	560	615	660	710
		14	707	435	490	513	570	625	685	743
Speed (m/min)	Boys	11	404	64	78	82	94	101	105	111
		12	387	65	78	84	95	105	110	117
		13	385	70	82	88	99	109	113	121
		14	295	76	88	94	105	114	118	125
	Girls	11	675	58	70	76	86	95	96	105
		12	767	65	75	83	90	100	105	115
		13	732	70	77	84	94	103	110	119
		14	707	73	82	86	95	104	114	124

**Fig. 1:**
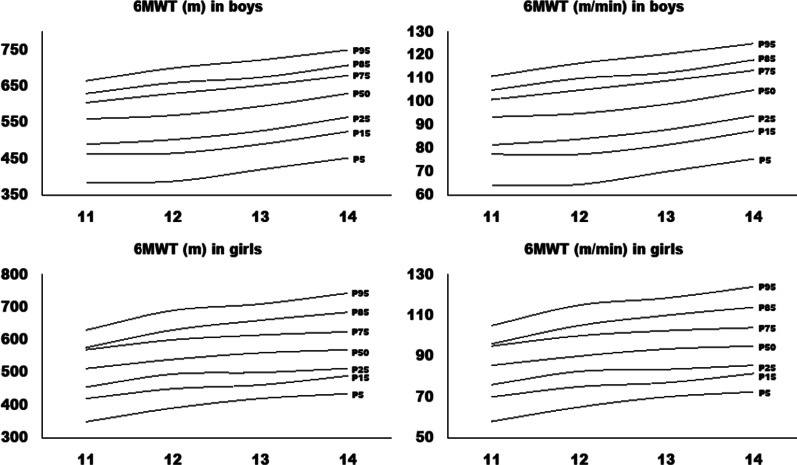
Sex- and age-specific normative data for distance covered (m) and mean walking speed (m/min) for the 6MWT (*N* = 4352)

Figure 1 shows sex- and age-specific normative data for distance covered and mean walking speed of the 6MWT. In general, the distance walked gradually increased by age, with the similar rate of change between ages in both sexes (*p* < 0.001). Boys in the specific age group performed better, compared to girls in the same age group.

## Discussion

The main purpose of the study was to create sex-specific and age-specific normative data for the 6MWT in a large sample of children aged between 11 and 14 years. The main findings of the study are: (1) boys performed better in the 6MWT, compared to girls, (2) older children performed better in the 6MWT, compared to their counterparts and (3) in the same age groups, boys outperformed girls in the 6MWT.

This is one of the first population-based studies aiming to establish normative data for the 6MWT in children. Our findings are comparable to previous studies conducted in different settings and with small sample sizes [2, 4, 5, 17–23]. Normative data for the 6MWT in healthy children have been reported in the United Kingdom [2], Taiwan [4], Turkey [5], United States [18], Saudi Arabia [19], Tunisia [20], Switzerland [21], Brazil [22], India [23] and Italy [26]. The mean value for distance covered in 6 min for the aforementioned studies ranged between 470 ± 59 m and 707 ± 102 [2, 20]. The findings of this study are mostly similar to normative data from Turkey [5], Saudi Arabia [19] and Brazil [22], while the shortest distance covered is observed in the United Kingdom [2], United States [18] and Tunisia [20]. The great range for the 6MWT of previous findings may be explained by the following parameters: (1) different age groups (from 4 to 17 years old [2, 21]), (2) sex distribution (boys and girls *vs*. only boys *vs*. only girls [19]), (3) convenience sample size without the calculation, (4) using different corridor length; i.e. from 15 to 20 m [2, 18], and (5) performing the test once or twice, with a 15, 30 or 60 min of interval [18, 20, 22] Moreover, a few studies have used physiological and biomechanical parameters, like heart rate, blood pressure, oxygen saturation or stride-length, which have all been associated with 6-min distance covered [12, 21]. Indeed, the normative data from previous studies must be taken with caution. First, the systematic review of Cacau et al. [17] has shown a great heterogeneity in distance covered for up to 159 m between the studies, pointing out that minimally clinically significant difference in children has not yet been established. Of note, such difference has been presented for adult population with cardiovascular and pulmonary diseases [27, 28]. Second, none of the study has used a sample size calculation, which makes difficult to generalize the findings to the whole population of children.

It has been well-documented, that the 6MWT is a reliable and valid measure to assess pulmonary capacities obtained from a submaximal level [1, 2, 17] Its practical implication has been highlighted in different populations, especially in those with disorders or postoperative stages [8–15] Although an effort for the 6MWT normative data in children has been made, this is the first study using a population-based sample and a sample size calculation to provide standards in Caucasian children between 11 and 14 years.

This study has a few strengths. We based the findings of the study on a large sample of 11–14-year-olds. The protocol for the 6MWT was followed according to the ATS Committee, to standardize the measuring procedure.

However, this study is not without limitation. First, by using a longitudinal design, we would be able to track biological changes in the 6MWT. Second, we did not collect the data regarding socioeconomic, health-related or biomechanical parameters. Previous studies have used heart rate, blood pressure, oxygen saturation, physical activity and stride-length as significant correlates of the 6MWT [12, 21]. Third, the distance covered was measured only once for each child, providing no information about the test–retest reliability for the studied sample. Fourth, the present study was unable to detect the level of motivation or coordination between and within the groups of children performing the 6MWT. Finally, the 6MWT wasn’t performed twice with a 30-min rest interval, as recommended by the ATS Committee [16]. Therefore, future research should be performed for each country to determine normative data for the 6MWT in the pediatric population, regarding test–retest reliability and validity properties (the associations between the 6MWT and health-related outcomes). Also, reference equations with sociodemographic, anthropological and health-related indicators for cross-correlation and predictive purposes need to be developed, in order to compare the data between the countries.

## Conclusions

This is the first population-based study providing sex-specific and age-specific normative data for the 6MWT in a large sample of apparently healthy children aged between 11 and 14 years. Our newly proposed normative data should serve in clinical and school-based settings; to screen for functional exercise capacity and to detect those individuals with ‘poor’ performance in the 6MWT. These individuals should be a ‘target group’ for special interventions and policies aiming to enhance the performance in the 6MWT.

## Data Availability

The datasets used and/or analyzed during the current study are available from the corresponding author on reasonable request.

## Abbreviations

ANOVA: Analysis of variance
L: Lambda
M: Mu
S: Sigma
SD: Standard deviation
6MWT: Six-minute walk test
95% CI: 95 Percent confident interval
